# Afpdb: an efficient structure manipulation package for AI protein design

**DOI:** 10.1093/bioinformatics/btae654

**Published:** 2024-11-05

**Authors:** Yingyao Zhou, Jiayi Cox, Bin Zhou, Steven Zhu, Yang Zhong, Glen Spraggon

**Affiliations:** Biologics Research Center, Novartis Biomedical Research, San Diego, CA 92121, United States; Biologics Research Center, Novartis Biomedical Research, San Diego, CA 92121, United States; Biologics Research Center, Novartis Biomedical Research, San Diego, CA 92121, United States; Metascape Foundation, Carlsbad, CA 92009, United States; Biologics Research Center, Novartis Biomedical Research, San Diego, CA 92121, United States; Biologics Research Center, Novartis Biomedical Research, San Diego, CA 92121, United States

## Abstract

**Motivation:**

The advent of AlphaFold and other protein Artificial Intelligence (AI) models has transformed protein design, necessitating efficient handling of large-scale data and complex workflows. Using existing programming packages that predate recent AI advancements often leads to inefficiencies in human coding and slow code execution. To address this gap, we developed the Afpdb package.

**Results:**

Afpdb, built on AlphaFold’s NumPy architecture, offers a high-performance core. It uses RFDiffusion's contig syntax to streamline residue and atom selection, making coding simpler and more readable. Integrating PyMOL’s visualization capabilities, Afpdb allows automatic visual quality control. With over 180 methods commonly used in protein AI design, which are otherwise hard to find, Afpdb enhances productivity in structural biology by supporting the development of concise, high-performance code.

**Availability and implementation:**

Code and documentation are available on GitHub (https://github.com/data2code/afpdb) and PyPI (https://pypi.org/project/afpdb). An interactive tutorial is accessible through Google Colab.

## 1 Introduction

The current wave of protein AI models revolutionizes protein design. An example design cycle involves diffusing backbone binder structures for a given target pocket using RFDiffusion (Watson *et al.* 2023), generating candidate sequences with ProteinMPNN (Dauparas *et al.* 2022), and prioritizing sequences based on predictions from AlphaFold 2 (Jumper *et al.* 2021). As current AI models are still in their infancy, a successful protein AI design campaign proposes thousands of candidates. A typical AI design workflow involves managing large-scale structure file read/write operations, performing structure alignment, measuring deviations, standardizing chain/residue labels, extracting residues, identifying mutations, and automating visualization generations. Existing programming packages provide solid strength and speed in various protein structure computations. These include, but are not limited to, Biopython (Hamelryck and Manderick 2003, Cock *et al.* 2009), pdb-tools (Rodrigues *et al.* 2018), pdbtools package (https://github.com/harmslab/pdbtools), BioPandas (Raschka 2017), Biotite (Kunzmann *et al.* 2023), Pyrosetta (Chaudhury *et al.* 2010), cctbx (Grosse-Kunstleve *et al.* 2002), gemmi (Wojdyr 2022), MDAnalysis (Michaud‐Agrawal *et al.* 2011), and ProDy (Bakan *et al.* 2011). Although they all enable the extraction of atom coordinates in Protein Data Bank (PDB) (Berman 2000) data files, these tools are often developed for lower-level data access or tailored toward specific applications such as molecular mechanics, dynamics, or crystallography. Their creation predates the recent protein AI breakthroughs, thus presenting a gap between the programming interface provided and the needs arising from AI design. This gap can result in requiring users to develop many lines of low-performance codes, which results in inefficiencies in both human coding and code execution (examples in Supplementary Note S1). The Python package Afpdb was created to address this opportunity. Biopython is used as the primary reference tool in this study, as it provides the most comprehensive programming interface for design tasks.

**Figure 1.**
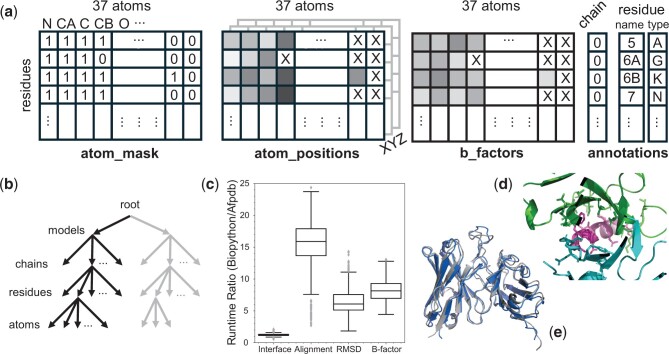
Architecture and performance comparison between Afpdb and Biopython. (a) Afpdb uses multi-dimensional NumPy arrays to represent a protein structure and key annotations; (b) Biopython represents molecular structures using a model-chain-residue-atom tree structure based on a container class called “PDB.Entity”; (c) Afpdb is consistently faster than Biopython, as benchmarked with four frequent computational tasks (*P* < 10^−30^ in all cases, one-side *t*-test, *n* = 633); (d) an example visualization of antibody-antigen binding interface generated with Afpdb’s “rs_around” and “PyMOL” methods; (e) comparison of the experimental (grey) and ESMFold-predicted (blue) F_ab_ domain of the 5CIL antibody.

Afpdb offers several advantages. First, Afpdb adopts a NumPy architecture, used by AlphaFold in its pre- and post-prediction phases (https://github.com/google-deepmind/alphafold) (Fig. 1a), to represent a protein structure. Biotite employs a comparable strategy. This data architecture not only simplifies access to atom coordinates, but also leverages the superb computational efficiency provided by NumPy’s C-language implementation to provide a high-performance implementation at its core. In comparison, Biopython utilizes a tree architecture (Fig. 1b) to comprehensively store protein structure data in a hierarchical format, which creates an overhead for extra user coding in coordinate extraction and subsequent structural measurement and transformation. Second, Afpdb adopts and extends the “contig” syntax and introduces several related classes. The generative AI model RFDiffusion introduces a succinct residue selection language called contig. Contig allows users to write code using the human-readable residue labels present in PDB files, instead of depending on potentially error-prone internal identifiers. This approach decreases boilerplate code and enhances both the readability and accuracy of the code. The “RL” class represents a list of residues in any order, the “RS” class handles deduplicated and unordered residues, and the “ATS” class facilitates atom type selection. Selections and their Boolean operations are important features found in PyMOL (Schrödinger LLC 2015) but missing in other tools. Third, users can combine the computational power of Afpdb with the outstanding 3D visualization strength of PyMOL by translating Afpdb selections into PyMOL “select” commands, allowing the automation of PyMOL commands through a seamless wrapper interface. In total, Afpdb implements over 180 methods driven by the protein AI design needs at our institute. Leveraging its feature-rich and high-performance implementations, Afpdb enables users to write less-but-faster code (Supplementary Note S1). We provide some examples in this study to highlight these strengths. Readers can find additional examples in the Jupyter Notebook tutorial and interactively experiment Afpdb with Google Colab. A real-life AI use case is presented here and more available online.

In summary, Afpdb’s NumPy architecture, contig adoption, and its PyMOL integration addresses the unmet needs in computational speed, coding friendliness, and visualization automation frequently encountered in AI protein design.

## 2 Results

### 2.1 Speed—acceleration by NumPy

AlphaFold 2 uses 2D and 3D NumPy arrays to model a protein structure (Fig. 1a), in contrast to Biopython’s model-chain-residue-atom tree structure (Fig. 1b). This avoids the overhead of using nested boilerplate loops to extract atom coordinates. Displacement calculations with the ready-to-operate NumPy arrays offer fast C-language implementations. In Afpdb, to represent coordinates of *n_a_* residues called group *a*, variable *atom_pos_a* would be of shape (*n_a_*, 37, 3), where 37 represents possible nonhydrogen atoms in a residue and 3 represents the *XYZ* coordinates. However, *atom_pos_a* can be reshaped into a 2D NumPy array *XYZ_a* of shape (*m_a_*, 3), where *m_a_* is the total number of unmasked atoms. A reference point, *point_c*, would be of shape (3), and the coordinates of another residue group, *b*, would be *XYZ_b* with shape (*m_b_*, 3). The displacement between atom group *a* and *point_c* can be computed in one fast NumPy minus operation resulting in an output array of shape (*m_a_*, 3):

XYZ_a – point_c

The displacement of all *m_a_* × *m_b_* atom pairs between group *a* and *b* can be computed in one step without looping:

XYZ_a[:, None] — XYZ_b[None,: ]

The output is a 3D array of shape (*m_a_*, *m_b_*, 3). Benchmark results using AbDb structures demonstrate the native NumPy representation offers a substantial speed increase compared to Biopython for certain critical structural computations (Fig. 1c, Supplementary Note S2), specifically, 16× for structural alignment, 6× for structural root mean square deviation (RMSD) calculation, and 8× for storing computed numerical properties on b-factors. A moderate 20% boost for interface residue identification was observed due to both implementations being highly optimized with a k-d tree acceleration. The speed gain does not require large memory consumption (Supplementary Note S3). With its internal high-performance core validated, we outline next the key Afpdb features that enable users to write efficient and concise code.

### 2.2 User-friendliness

Afpdb follows the design principle of reducing methods and arguments whenever possible. For example, reading/writing protein structures in different formats can be done with only two methods:

p = Protein('5cil') # read from PDB online

p.save('5cil.cif') # save to a local .cif file

q = Protein('5cil.cif') # read the local .cil file

# AlphaFold model for human TLR7 by UniProt ID

s = Protein('Q9NYK1')

s.save('TLR7.pdb') # save to a local .pdb file

Implementing a similar use case with Biopython requires five methods, PDBList.retrieve_pdb_file, PDBParser.get_structure, MMCIFParser.get_structure, PDBIO.save, and MMCIFIO.save, depending on the input/output file formats. Biopyhon and other tools do not yet support accessing public AI model repositories (https://alphafold.ebi.ac.uk/). PDB entry 5CIL will be used in the remaining examples.

### 2.3 Contig—a powerful residue selection language

Afpdb extends the contig syntax to enable easy residue selection and intuitive Boolean combinations. Residue selection is a noticeably convenient feature of PyMOL that is missing from Biopython and other tools. Here we show an example of selecting the interface residues between the antibody (chain H and L) and the antigen (chain P):

rs_HL, rs_P, dist = p.rs_around('P', rs_within = 'H:L', dist = 4)

rs_both = rs_HL | rs_P

print(rs_both)

# output: H28,31-35,47,50-52,53-54,56-58,95,100C-100F,100H,100J:L30,32,91-96:P671-677,679-680,683

q = p.extract(rs_both, ats = 'N,CA,C,O')

The example illustrates rs_around method identifies H/L interface residues with respect to the antigen (chain P), which can be union-combined with interface residues on the antigen into a new residue selection object called rs_both (40 residues in total). The output string is the contig representation of rs_both. This printed example string demonstrates the basic syntax of the contig language, including a single residue fragment (residue 28) on chain H, a 4-residue fragment (100C-100F) on chain H, and contigs from different chains concatenated with colons. Afpdb handles residue insertion codes (e.g. “100C”) not supported in RFDiffusion and the original AlphaFold code base. The last line shows how a backbone substructure can be extracted with the residue selection rs_both and an atom selection “N,CA,C,O.” If we use pdb-tools for such extraction, it will require both pdb_selchain and pdb_selatom command line tools, but it does not support such granular selection at the residue-level. Employing contig eliminates the need to list residues and atoms through loops, thereby greatly minimizing human coding effort and errors.

### 2.4 PyMOL—a bridge for 3D visualization

The following example shows how we can leverage the computational power of Afpdb to identify interface residues, then translate the residue selection into a PyMOL selection command, and finally using the native PyMOL commands to automate the generation of a 2D image (Fig. 1d) and 3D PyMOL session file for viewing the atomic details of the binding interface. Combining the unique strength of Afpdb and PyMOL provides the feasibility of high-throughput visual quality controls on the thousands of intermediate candidates during an AI protein design campaign.

rs = rs_both.str(format = 'PYMOL', rs_name = 'myint')

# rs string is: 'select myint,(chain H and resi 28 + 31-35 + 47+50-51 + 52+53-54 + 56-58 + 95+100C+100D+100E+100F+100H+100J)

or (chain L and resi 30 + 32+91-96) or (chain P and resi 671-677 + 679-680 + 683)'

cmd = f'''fetch 5cil, myobj; remove solvent; util.cbc;

{rs}

show sticks, myint; zoom myint; deselect;

save myint.png; save myint.pse'''

Protein.PyMOL().run(cmd)

Alternatively, 3D structure visualization within Jupyter Notebooks is enabled through Afpdb’s integration with Py3DMol (https://github.com/avirshup/py3dmol), as illustrated in the tutorial.

### 2.5 AI use case—protein structure prediction and evaluation

In the example, Afpdb automates structure prediction with ESMFold web service (https://esmatlas.com/). Internally, it produces one sequence by concatenating chains with poly-glycine linkers and replacing any missing residues with glycine. Inserted residues were removed from the prediction structure upon return. The RMSD between experimental and predicted structures, after alignment, is 2.46 Å in this example (Fig. 1e).

# extract Fv-only, ESMFold has limits on sequence length

exp = Protein("5cil").extract("H-113:L-110")

# predicted structure from sequence with ESMFold service

pred = p.fold(exp.seq())

#aligntwo backbones, then measure RMSD, output 2.46A

rmsd = pred.rmsd(exp, ats = "N,CA,C,O", align = True)

More sophisticated real-life AI use cases highlighting how Afpdb facilitates protein designs are described in the tutorial. Examples include using Afpdb to pre- and post-process missing residues for AlphaFold, automating protein structure prediction using the ESMFold web service, and creating full-atom models for proteins designed with RFDiffusion and ProteinMPNN. Afpdb’s implementation of binding scores for EvoPro’s (Goudy *et al.* 2023) is fifteen times more concise and significantly faster by two orders of magnitude (Supplementary Note S1). Lastly, Afpdb provides parsers for easy interfacing output files generated by AI models, including AlphaFold, ColabFold (Mirdita *et al.* 2022), ESMFold, ProteinMPNN, and LigandMPNN (Dauparas *et al.* 2023).

### 2.6 Limitations

Inheriting the power of AlphaFold’s NumPy architecture also comes with limitations. The arrays use residues as rows and 37 nonhydrogen atoms as columns, constraining Afpdb to represent only standard protein structures without annotation metadata. Afpdb cannot represent post-translation modifications, unnatural amino acids, as well as compounds, RNA (ribonucleic acid), and DNA (Deoxyribonucleic acid) contained in the PDB file. These additional molecular structure types are now supported in emerging AI models such as AlphaFold3 (Abramson *et al.* 2024) and RoseTTAFold All-Atom (Krishna *et al.* 2024). However, the many successes of the protein-only AI models (Watson *et al.* 2023, Bennett *et al.* 2024) demonstrate there remains a rich set of AI applications, including mini-protein design and antibody design, that can directly benefit from Afpdb. The release of Afpdb as an open-source software provides the opportunity for the community to expand Afpdb into supporting additional molecule types while preserving its large set of productivity features.

## Author contributions

Yingyao Zhou (Conceptualization, Software, Formal analysis, Supervision, Visualization, Writing—original draft, Writing—review & editing), Jiayi Cox (Software, Writing—review & editing), Bin Zhou (Software, Writing—review & editing), Steven Zhu (Software, Writing—review & editing), Yang Zhong (Software, Writing—review & editing), and Glen Spraggon (Supervision, Writing—review & editing)

## Supplementary Material

btae654_Supplementary_Data

## Data Availability

The source code and benchmarking results can be accessed at https://doi.org/10.6084/m9.figshare.27018589.v1.

## References

[btae654-B1] Abramson J , Adler J, Dunger J et al Accurate structure prediction of biomolecular interactions with AlphaFold 3. Nature 2024;**630**:493–500. 10.1038/s41586-024-07487-w PMC11168924 38718835

[btae654-B2] Bakan A , Meireles LM, Bahar I. ProDy : protein dynamics inferred from theory and experiments. Bioinformatics 2011;27:1575–7. 21471012 10.1093/bioinformatics/btr168 PMC3102222

[btae654-B3] Bennett NR , Watson JL, Ragotte RJ et al Atomically accurate de novo design of single-domain antibodies. bioRxiv, 2024.

[btae654-B4] Berman HM . The protein data bank. Nucleic Acids Res 2000;28:235–42. 10592235 10.1093/nar/28.1.235 PMC102472

[btae654-B5] Chaudhury S , Lyskov S, Gray JJ. PyRosetta: a script-based interface for implementing molecular modeling algorithms using Rosetta. Bioinformatics 2010;26:689–91. 20061306 10.1093/bioinformatics/btq007 PMC2828115

[btae654-B6] Cock PJA , Antao T, Chang JT et al Biopython: freely available Python tools for computational molecular biology and bioinformatics. Bioinformatics 2009;25;1422–3. 19304878 10.1093/bioinformatics/btp163 PMC2682512

[btae654-B7] Dauparas J , Anishchenko I, Bennett N et al Robust deep learning-based protein sequence design using ProteinMPNN. Science 2022;378:49–56. 36108050 10.1126/science.add2187 PMC9997061

[btae654-B8] Dauparas J , Lee GR, Pecoraro R et al Atomic context-conditioned protein sequence design using LigandMPNN. biorXiv, 10.1101/2023.12.22.573103, 2023, preprint: not peer reviewed. PMC11978504 40155723

[btae654-B9] Ferdous S , Martin ACR. AbDb: antibody structure database-a database of PDB-derived antibody structures. Database (Oxford) 2018;2018:bay040. 10.1093/database/bay040 PMC5925428 29718130

[btae654-B10] Goudy OJ , Nallathambi A, Kinjo T et al In silico evolution of autoinhibitory domains for a PD-L1 antagonist using deep learning models. Proc Natl Acad Sci USA 2023;120:e2307371120. 10.1073/pnas.2307371120 PMC10710080 38032933

[btae654-B11] Grosse-Kunstleve RW , Sauter NK, Moriarty NW et al The computational crystallography toolbox : crystallographic algorithms in a reusable software framework. J Appl Crystallogr 2002;35:126–36.

[btae654-B12] Hamelryck T , Manderick B. PDB file parser and structure class implemented in Python. Bioinformatics 2003;19:2308–10. 14630660 10.1093/bioinformatics/btg299

[btae654-B13] Jumper J , Evans R, Pritzel A et al Highly accurate protein structure prediction with AlphaFold. Nature 2021;596:583–589. 34265844 10.1038/s41586-021-03819-2 PMC8371605

[btae654-B14] Krishna R , WangJ, AhernW et al Generalized biomolecular modeling and design with RoseTTAFold All-Atom. Science2024;384:eadl2528.38452047 10.1126/science.adl2528

[btae654-B15] Kunzmann P , Müller TD, Greil M et al Biotite: new tools for a versatile Python bioinformatics library. BMC Bioinformatics 2023;24:236. 37277726 10.1186/s12859-023-05345-6 PMC10243083

[btae654-B16] Michaud‐Agrawal N , Denning EJ, Woolf TB et al MDAnalysis: a toolkit for the analysis of molecular dynamics simulations. J Comput Chem 2011;32:2319–27. 21500218 10.1002/jcc.21787 PMC3144279

[btae654-B17] Mirdita M , Schütze K, Moriwaki Y et al ColabFold: making protein folding accessible to all. Nat Methods 2022;19:679–82. 35637307 10.1038/s41592-022-01488-1 PMC9184281

[btae654-B18] Raschka S. BioPandas: working with molecular structures in pandas DataFrames. J Open Source Softw 2017;2:279.

[btae654-B19] Rodrigues JPGLM , Teixeira JMC, Trellet M et al pdb-tools: a swiss army knife for molecular structures. F1000Res 2018;7:1961. 30705752 10.12688/f1000research.17456.1 PMC6343223

[btae654-B20] Schrödinger LLC. *The PyMOL Molecular Graphics System, Version∼1.8*. Pymol, 2015.

[btae654-B21] Watson JL , Juergens D, Bennett NR et al De novo design of protein structure and function with RFdiffusion. Nature 2023;620:1089–100. 37433327 10.1038/s41586-023-06415-8 PMC10468394

[btae654-B22] Wojdyr M. GEMMI: a library for structural biology. J Open Source Softw, 2022;7:4200.

