# Supplementary material for: Afpdb: an efficient structure manipulation package for AI protein design
Source: Bioinformatics. 2024 Nov 5;40(12):btae654. doi: 10.1093/bioinformatics/btae654 (PMC11631506; doi:10.1093/bioinformatics/btae654)
Supplement: btae654_Supplementary_Data [file btae654_supplementary_data.pdf]

Supplementary Notes

Afpdb – an efficient structure manipulation package for AI protein design

Yingyao Zhou, Jiayi Cox, Bin Zhou, Steven Zhu, Yang Zhong, Glen Spraggon

Note 1. Code snippets illustrating how Afpdb accelerates coding

Afpdb is not intended to replace established protein structure manipulation packages. Instead, it introduces new methods absent from existing Python tools that predate protein AI technologies. Without Afpdb, users would need to write significantly more code. Supplementary Table 1 compares the lines of Python code required to accomplish various computational tasks. We first compare four tasks, as outlined in Figure 1.c, using code from our Afpdb and Biopython (Cock *et al.*, 2009) implementations in the benchmark script described in Supplementary Note 2. Tasks that can be completed with Afpdb in one or two lines of method calls require approximately 7-45 lines of code without Afpdb. Additionally, we compare our Afpdb-based implementations to community code for two real-life AI-related tasks: computing the EvoPro binding score and the DockQ score. Afpdb reduces the coding effort by 94% and 60% for EvoPro (Goudy *et al.*, 2023) and DockQ (Basu and Wallner, 2016) computations, respectively. While Afpdb may not be indispensable for AI design tasks, the comparison table indicates that it can considerably cut down on human coding time, thus enhancing readability.

Supplementary Table 1. Estimated lines of code required for various computational tasks using equivalent input and output data.

Example	Without Afpdb[*]	With Afpdb[*]
Identify interface residues	45[+]	2[+]
Align two protein structures	16[+]	1[+]
RMSD of two structures	7[+]	1[+]
Assign B-factors	9[+]	1[+]
EvoPro Contact Score	67[#]	4[@]
DockQ Score	134[&]	54[$]

* Codes written by users as separate methods but utilized by the main method is counted. Pre- and post-processing code, empty lines, print statement, and comment lines are excluded. Extra syntax lines due to coding styles are not double counted in the community code.

\+ Benchmark script (https://github.com/data2code/afpdb/blob/main/benchmark/benchmark_script.py)
\# Method score_binder_complex (https://github.com/Kuhlman-Lab/evopro)
@ Afpdb AI Use Case 4 (https://github.com/data2code/afpdb/blob/main/tutorial/Afpdb_Tutorial.pdf)
& Method calc_DockQ (https://github.com/bjornwallner/DockQ/blob/master/src/DockQ/DockQ.py)
$ Afpdb source code, method dockQ.

Both EvoPro and DockQ examples require the identification of contact residues and their distances. Although Biopython provides programming access to atom coordinates, it lacks a method for this specific task.
Afpdb's rs_around method accomplishes it with a single method call. In contrast, EvoPro's implementation uses the following algorithm to identify contact residue pairs:

```
for residue_p in protein_a
    for residue_q in protein_b
        for atom_i in residue_p:
            for atom_j in residue_q:
                compute_atom_distance(atom_i, atom_j)
        compute_residue_distance(residue_p, residue_q)
```

The four nested loops in Python can be computationally expensive. Our benchmark shows EvoPro's version is, on average, 289 times slower compared to Afpdb's solution (details in Supplementary Note 2).

DockQ improves performance using the following algorithm:

```
XYZ_a, n_atom_per_res_a = atom_coordinate(protein_a)
XYZ_b, n_atom_per_res_b = atom_coordinate(protein_b)
atom_dist = np.sqrt(np.sum(XYZ_a[:, None] - XYZ_b[None, :])**2, axis = -1))
compute_res_distance(atom_dist, n_atom_per_res_a, n_atom_per_res_b)
```

The first two lines convert atom coordinates stored in Biopython's tree structure into a more computing-friendly Numpy array format (see the get_residue_distances method in the DockQ.py file), aligning with Afpdb's data architecture. The third line leverages Numpy's speed, similar to Afpdb's implementation. However, the last line contains two nested loops inside, making this implementation, on average, 2.2 times slower than Afpdb's. DockQ also offers a Cython implementation (https://cython.org/), adding approximately 25 more lines of code using C-language syntax to further accelerate execution (see the residue_distances method in the operations.pyx file). However, this advanced technique is less accessible to many users and was not used in EvoPro's implementation.

In addition to identifying contact residues, Afpdb's contig syntax and residue/atom selection objects simplify the remaining DockQ logic, where aligned atom pairs are prepared and RMSD is computed (see the dockQ method in Afpdb's source code). The implementation of the DockQ score exemplifies the benefits Afpdb provides, as this multi-component computational task is frequently used in AI protein design tasks.

Notes 2. Compare running time between Afpdb and Biopython

Performance and memory benchmarking utilized 633 antibody-antigen structures from the non-redundant antibody structure database (AbDb, http://www.abybank.org/abdb/) (Ferdous and Martin, 2018), with each structure averaging 414 amino acids and comprising 3 chains. The alignment and RMSD calculation tests require an input of a structure pair, wherein the second structure is generated using ColabFold (Mirdita *et al.*, 2022). All source code, input structures, and sample outputs are available at (https://doi.org/10.6084/m9.figshare.27018589.v1).

To run the benchmark, use the following command:

python benchmark_script.py

The script will generate an output file named afpdb_cost.csv. A sample output is provided for reference.

Running time for Biopython was measured only on the core computing steps, with the code carefully optimized. Running times for each structure were collected, ratios derived, and used to create the box plot in Figure 1.C. Specifically, the running time for each input structure is measured as the sum of 20, 100, 500, and 500 repeats for the interface, alignment, RMSD, and B-factor use cases, respectively, as described in Figure 1.C. Results in Figure 1.C were obtained using Python 3.9.16, NumPy 1.23.5, Pandas 1.5.3, and Biopython 1.81. The server runs Red Hat Enterprise Linux release 8.8 with an AMD EPYC 7513 32-Core Processor and 1TB RAM. However, the hardware and operating system should not affect the benchmark results, as all packages were used on the same system and only the ratio of the running time is considered.

To compare the running time of the contact residue identification algorithm employed in EvoPro, DockQ, and Afpdb, we utilized a consistent set of 633 structures, repeating the process 5 times. The script used is loop_script.py, and our sample output file is named afpdb_dist.csv.

Note 3: Memory usage profiling of Afpdb and Biopython

Using the same AbDb dataset, we profile memory usage with the following command:

python memory_script.py

This script generates an output file named afpdb_mem.csv. For reference, we offer a sample output gathered from the same server mentioned in Supplementary Note 2.

For measuring memory usage after task completion, we utilize psutil.Process.memory_info.rss in Python. To gauge the peak intermediate memory used during task execution, we employ Memory Profiler (https://pypi.org/project/memory-profiler).

Afpdb consumes more memory upon library import -- 151 MB compared to Biopython's 42 MB. This is because Afpdb imports pymol2 (https://www.pymol.org), py3Dmol (https://github.com/avirshup/py3dmol), and selective modules from AlphaFold (Jumper *et al.*, 2021), ColabFold, and ColabDesign (https://github.com/sokrypton/ColabDesign) packages to support AI and visualization features. This initial memory usage remains constant regardless of the number or size of structures processed. As anticipated, Afpdb slightly increases the memory needed to store a typical protein structure, utilizing 11 ± 4 MB with Numpy arrays versus 9 ± 3 MB with Biopython's compact tree format.

The maximum dynamic memory required for computational tasks, as detailed in Figure 1.c, is more relevant. Profiling shows that Afpdb uses about 29-56% of the memory that Biopython requires in these cases (Supplementary Figure 1). One explanation is that Biopython's tree-format data often must be extracted and reformatted into a more computationally efficient array format, increasing its dynamic memory usage. This strategy is observed in the DockQ community code.

The memory consumption of either Afpdb or Biopython should not be a major concern for typical protein design tasks, given that most users have access to tens of GBs of memory.

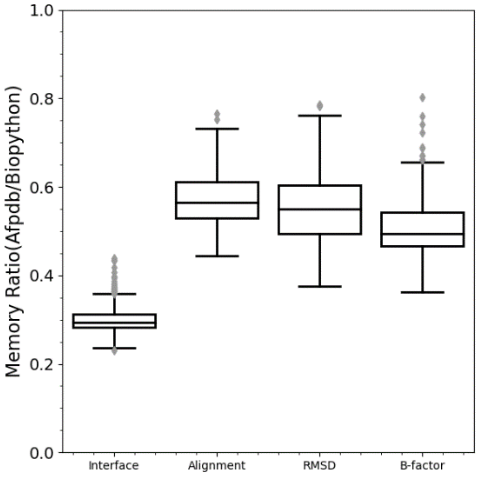

Supplementary Figure 1. Analysis of peak memory usage between Biopython and Afpdb. Afpdb exhibits lower peak memory consumption compared to Biopython, as demonstrated in four common computational tasks ($p < 10^{-30}$ in all cases, one-side *t*-test, $n = 633$).

References

Basu,S. and Wallner,B. (2016) DockQ: A Quality Measure for Protein-Protein Docking Models. *PLoS One*, **11**, e0161879.

Cock,P.J.A. *et al.* (2009) Biopython: freely available Python tools for computational molecular biology and bioinformatics. *Bioinformatics*, **25**, 1422–3.

Ferdous,S. and Martin,A.C.R. (2018) AbDb: antibody structure database-a database of PDB-derived antibody structures. *Database (Oxford)*, **2018**.

Goudy,O.J. *et al.* (2023) In silico evolution of autoinhibitory domains for a PD-L1 antagonist using deep learning models. *Proceedings of the National Academy of Sciences*, **120**.

Jumper,J. *et al.* (2021) Highly accurate protein structure prediction with AlphaFold. *Nature*, **596**, 583–589.

Mirdita,M. *et al.* (2022) ColabFold: making protein folding accessible to all. *Nat Methods*, **19**, 679–682.
